# Do Laser-Activated Irrigation Protocols Improve Endodontic Success? A Prospective Clinical Comparison of 1-Year Periapical Healing with Sonic, Ultrasonic, Manual Dynamic and Conventional Techniques

**DOI:** 10.3390/diagnostics16071003

**Published:** 2026-03-26

**Authors:** Medine Çiçek, Ahter Şanal Çıkman, Dilara Nil Günaçar

**Affiliations:** 1 Department of Endodontics, Faculty of Dentistry, Recep Tayyip Erdoğan University, 53100 Rize, Turkey; ahter.sanalcikman@erdogan.edu.tr; 2 Department of Oral and Maxillofacial Radiology, Faculty of Dentistry, Recep Tayyip Erdogan University, 53100 Rize, Turkey; dilaranil.tomrukcu@erdogan.edu.tr

**Keywords:** fractal analysis, irrigation activation, PAI, PIPS, SWEEPS

## Abstract

**Background**: Successful healing of chronic apical periodontitis after endodontic treatment requires a reduction in the size of the radiolucent area and the healing of the bone. This study aimed to compare the effects of different irrigation activation techniques on healing in single-rooted mandibular premolar teeth with periapical lesions of endodontic origin. **Methods**: A total of 132 systemically healthy patients with mandibular single-rooted premolar teeth and a periapical index (PAI) score ≥ 3 were assigned to five experimental groups (Sonic activation, Passive ultrasonic irrigation, Photon-Induced Photoacoustic Streaming, Shock Wave Enhanced Emission Photoacoustic Streaming and Manual dynamic activation) and a control group (Conventional Syringe Irrigation). After access cavity preparation, the canals were prepared up to three sizes larger than the initial apical diameter with 5 mL of 2.5% NaOCl used between each file. Final irrigation was performed via the assigned activation system. The root canals were obturated with gutta-percha in a single visit. The effects of the activation systems on healing were compared at 1-year follow-up. The primary outcome measure was the change in lesion diameter. PAI score and fractal dimension (FD) were evaluated as secondary outcomes. **Results**: At the 1-year follow-up, FD values significantly increased, PAI scores and lesion size decreased in all groups compared with baseline (*p* < 0.001). However, the increase in FD was comparable among the irrigation groups (*p* > 0.05). In contrast, lesion size reduction and PAI-based healing rates favored the laser-activated groups. The PAI scores and lesion size in the control group were significantly greater than that in the laser groups (*p* < 0.05). **Conclusions**: At the 1-year follow-up, all the groups presented similar FD increases, while the laser irrigation groups presented significantly greater reductions in lesion size than did the control group.

## 1. Introduction

The primary goal of endodontic treatment is to treat the infection by optimally cleaning the root canal system and preventing reinfection [1,2]. Given the complex anatomy of the root canal system, mechanical cleaning alone is not sufficient to completely eliminate bacteria from the root canal [3]. Root canal preparation must be accompanied by an effective irrigation activation technique (IAT) to effectively remove foci of infection [1,2].

During mechanical instrumentation, a layer called the “smear layer” forms on the walls of the root canal [2]. Composed of organic and inorganic materials, its removal is crucial [1,4]. Because, it hinders irrigants and filling materials from penetrating the dentinal tubules, making disinfection more difficult [4]. Therefore, various irrigant solutions are used to enhance root canal disinfection and reduce bacterial load [1,2,5]. One commonly used material is sodium hypochlorite (NaOCl) [1,2,6]. Despite its high toxicity and inability to remove the inorganic component of the smear layer, it is widely used due to its antibacterial properties and ability to dissolve necrotic tissue [1,2].

The irrigant solutions are typically delivered into the root canal via a syringe and needle [1,7]. However, conventional syringe irrigation (CSI) has limitations related to needle tip insertion, an increased risk of apical extrusion of the irrigant and its inability to effectively clean areas not reached by mechanical instrumentation [1,7,8]. Therefore, various techniques have been developed to enhance the penetration and distribution of irrigant solutions throughout the root canal system [1,2].

Manual dynamic activation (MDA) is an IAT performed by up and down movements of a gutta-percha compatible with the final instrumentation size. This movement creates different pressure zones in the canal, allowing the irrigant to contact more surfaces [9,10].

Sonic activation (SA) primarily relies on low-frequency vibration to move irrigating solutions through fluid dynamics, creating a strong hydrodynamic phenomenon and increase the effectiveness of the solution by using flexible tips connected to special instruments [11]. Passive ultrasonic irrigation (PUI) is another IAT based on the transmission of acoustic energy to the irrigant in the canal through a vibrating file or a thin wire with hydrodynamic activation [2,12]. It contributes to disinfection by inducing cavitation of the solution in the canal and increasing the contact of the solution with inaccessible surfaces [2].

Recently, some laser activation methods have been developed as photon-initiated photoacoustic flow (PIPS) and shock wave-enhanced emission photoacoustic flow (SWEEPS) [13,14]. In these methods, a special fiber tip is placed in the access cavity to deliver the laser energy [14]. PIPS activates the irrigant using low-energy Er:YAG laser pulses, inducing rapid vapor bubble formation. The expansion and collapse of these bubbles generate photoacoustic shock waves, enabling three-dimensional movement of the solution throughout the root canal [14]. The SWEEPS technique, developed to enhance the cleaning and disinfection efficacy of PIPS, differs from PIPS by applying a dual-pulse emission to the irrigant, which accelerates bubble collapse [15]. The mechanism of SWEEPS relies on the emission of several consecutive laser pulses with the second pulse delivered during the final stage of collapse of the bubble created by the first pulse. This generates powerful shock waves, allowing the irrigant to penetrate deeper into narrow root canals [14,15].

Apical periodontitis (AP) is a common oral inflammatory disease characterized by alveolar bone resorption in the periapical tissues [16]. A recent study reported that approximately half of the global adult population has AP in at least one tooth [17]. In a recent meta-analysis, an increase in the prevalence of AP in the adult general population was reported in both root canal treated and untreated teeth [18]. It is well established that the persistence of bacteria within the root canal system contributes to chronic inflammation of the periapical tissues in AP [16], a condition that frequently manifests as asymptomatic chronic apical periodontitis (CAP) [19]. Successful healing of AP requires a reduction in the size of the radiolucent area and healing of the bone [16].

The periapical index (PAI) system, which grades periapical pathology from 1 to 5 according to increasing radiographic appearance, helps to achieve consensus among clinicians in categorizing lesions. PAIs 1 and 2 are categorized as ‘healed’, and PAIs 3, 4 and 5 are categorized as ‘unhealed’ [20]. Periapical lesions can only be visually detected on conventional radiographs after approximately a 30% alteration in bone mineralization occurs [21]. Although these imaging techniques allow a macroscopic evaluation of the healing process, provide limited information regarding the micromorphology of bone and its complex structural organization [22].

Recent studies have shown that the trabecular microstructure of the bone should also be considered when evaluating bone density [23,24]. Fractal analysis (FA) is a quantitative method for assessing changes in bone tissue [23]. It is widely used in radiographs to detect and evaluate changes in bone, apical healing, periapical bone, and systemic conditions affecting bone [23,24].

Numerous studies have evaluated the effectiveness of smear layer and debris removal to compare the cleaning efficiency of different IATs; however, the results are inconsistent [25,26,27,28,29,30]. Some studies have reported that SWEEPS is superior to CSI in removing debris [25], eliminating the smear layer in cases with fractured instruments [26], and removing pulp tissue remnants in isthmus areas [27], and that SWEEPS removes a larger amount of debris compared to PIPS [28]. However, other studies have reported that PIPS and SWEEPS exhibit similar efficacy in reducing bacterial load [29] and in smear layer removal [30]. However, there are only a limited number of studies investigating the effects of IATs on periapical lesion healing [31,32]. In an in vivo study including incisors and single-rooted premolars with CAP, laser-activated irrigation and PUI were reported to enhance endodontic treatment success compared with CSI [31]. In a rat model of AP, PUI and SA were associated with smaller periapical lesions compared with the control and CSI groups. Furthermore, SA was shown to significantly reduce both lesion size and the inflammatory response in the periapical region [32].

In order to contribute to the conflicting results in the existing literature, this study aimed to compare the effects of different IATs (MDA, SA, PUI, PIPS, SWEEPS) on healing in single-rooted mandibular premolar teeth with endodontic periapical lesions.

The null hypothesis of this study was that there would be no difference in lesion healing among the IATs.

## 2. Materials and Methods

This study was conducted and reported in accordance with the TREND statement, and the completed TREND checklist is provided in Supplementary Table S1. The study population consisted of patients who applied to Recep Tayyip Erdoğan University (Rize, Turkey). Local ethics committee approval was obtained from the Recep Tayyip Erdoğan University Ethical Committee (No:2023/136). The study protocol was registered at ClinicalTrials.gov (ID: NCT06991803). All the participants were informed about the study protocol and written informed consent was obtained.

### 2.1. Sample Size Calculation

The G Power 3.1.9.4 (University Kiel, Kiel, Germany) program was used to calculate the effect size. The effect size was calculated on the basis of the data of Verma et al. [31], who compared the success of different irrigation techniques in healing after one year. On the basis of the chi-square test data, an effect size of 0.388 was found to be sufficient for significance, and it was calculated that a total of at least 110 samples were required with a type 1 error of 0.05 and 90% power. At least 18 samples are required for each group. However, considering potential data loss, 22 samples were used for each group.

### 2.2. Patient Selection and Allocation

Vertucci Class I single-rooted mandibular premolars with asymptomatic AP and PAI score of 3 or higher were included in the study. Patients with systemic diseases, bone metabolism diseases and/or drugs that affect bone metabolism (steroids and bisphosphonates) were excluded from the study. Immunocompromised patients, patients with a history of radiotherapy, pregnant patients, teeth with Miller 2 or more mobility, teeth with a periodontal pocket depth of ≥5 mm, teeth with internal and external resorption, and teeth with vertical and horizontal root fractures were excluded.

Out of 150 patients aged 18 years and older, 6 did not meet the study criteria and 12 refused to participate. Therefore, 132 patients were included and assigned to six groups (five irrigation activation groups and a control group), with 22 patients per group. To ensure a balanced distribution of patients requiring root canal treatment (RCT) and retreatment (RT) across the groups, a quasi-randomization method was applied. Patients were sequentially and cyclically allocated based on their enrollment order. For RCT cases, the first patient was assigned to Group 1, the second to Group 2, and so on, cycling through the groups until all RCT patients were allocated. The same procedure was applied for RT cases.

Control Group: CSI (n = 22; 11 RCT, 11 RT);
Group 1: MDA (n = 22; 10 RCT, 12 RT);
Group 2: SA (n = 22; 12 RCT, 10 RT);
Group 3: PUI (n = 22; 11 RCT, 11 RT);
Group 4: PIPS (n = 22; 12 RCT, 10 RT);
Group 5: SWEEPS (n = 22; 10 RCT, 12 RT).

### 2.3. Clinical Procedure

Preoperative panoramic radiographs (OPGs) were obtained with a Planmeca Promax 2D S2 device (Planmeca Romexis, Helsinki, Finland). The patients were positioned so that the sagittal plane was parallel to the vertical plane of the dental panoramic machine and the Frankfurt plane was parallel to the floor. The same radiographic exposure settings (66 kVp, 8 mA and 16.6 s exposure time) were used for all patients. For each tooth, the vertical, horizontal and diagonal dimensions passing through the center of the lesion were measured via ImageJ v1.52 software (National Institutes of Health, Bethesda, MD, USA), and the largest dimension obtained was recorded as the preoperative lesion diameter [33]. The program was downloaded from the internet at https://imagej.nih.gov/ij/download.html (accessed on 13 June 2024).

After local anesthesia was applied, the teeth were isolated with a rubber dam and the endodontic access cavity was opened with a sterile diamond rond bur under water cooling. Then, #10–15 K-type hand files (Dentsply Maillefer, Ballaigues, Switzerland) were inserted into the canals, and after determining point 0.0 with the Root ZX mini electronic apex locator (J. Morita Co., Tokyo, Japan) according to the manufacturer’s instructions, the working length was determined to be 0.5 mm shorter than this point and confirmed radiographically. When a discrepancy was observed, the apex locator was considered correct.

After the initial apical diameter with the largest K-type file trapped in the working length was determined, the root canals were prepared with ProTaper Next (Dentsply Maillefer, Ballaigues, Switzerland) up to 3 sizes larger than the initial diameter via a torque-controlled endodontic motor (SybronEndo, Glendora, CA, USA) in 300 rpm/2–5.2 Ncm rotation mode. Between each file, the canals were irrigated with 5 mL of 2.5% NaOCl (Senem Dental, Kayseri, Türkiye). In RT cases, after opening the access cavity under rubber dam isolation, the gutta-percha was removed with RT files (EndoArt RT, İnci Dental, Istanbul, Türkiye), and the rest of the procedure was performed in the same manner as for primary RCT.

#### 2.3.1. Control Group (CSI)

In this group, the CSI was used for final irrigation of the root canals. The canals were irrigated with 17% EDTA solution (Saver, Prime Dental, Izmir, Türkiye) in three consecutive 20 s applications of 2 mL each, with fresh solution used for each period, totaling 6 mL over 1 min. Between EDTA and NaOCl irrigation, 2 mL of saline was applied for 20 s to prevent chemical interaction. 2.5% NaOCl was then applied in three consecutive 20 s applications of 2 mL each, again using fresh solution for each period, totaling 6 mL over 1 min. A 30-gauge perforated irrigation needle (Berika Dental, Konya, Türkiye) was placed 1–2 mm short of the working length, and during irrigation, 1–2 mm up-and-down movements were performed with constant low pressure, according to previously described protocols in the literature [10].

#### 2.3.2. Group 1 (MDA)

After the root canal was filled with the irrigant solution, a gutta-percha cone compatible with the master file was positioned 1 mm short of the working length and moved in an up-and-down motion at 100 strokes/minute for activation, according to previously described protocols in the literature [10,28]. The canals were irrigated with 17% EDTA in three consecutive 20 s applications of 2 mL each, with fresh solution used for each period, totaling 6 mL over 1 min. Between EDTA and NaOCl irrigation, 2 mL of saline was applied for 20 s to prevent chemical interaction. 2.5% NaOCl was then applied in three consecutive 20 s applications of 2 mL each, again using fresh solution for each period, totaling 6 mL over 1 min.

#### 2.3.3. Group 2 (SA)

SA was performed using the Easydo Activator device (EA; Easyinsmile, Changsha, China). While the solution was present in the canal, the polymer tip (Easyinsmile, Changsha, China) was placed 2 mm short of the determined working length, and the irrigant was activated at the recommended power setting according to the manufacturer’s instructions. The canals were irrigated with 17% EDTA in three consecutive 20 s applications of 2 mL each, with fresh solution used for each period, totaling 6 mL over 1 min. Between EDTA and NaOCl irrigation, 2 mL of saline was applied for 20 s to prevent chemical interaction. 2.5% NaOCl was then applied in three consecutive 20 s applications of 2 mL each, again using fresh solution for each period, totaling 6 mL over 1 min.

#### 2.3.4. Group 3 (PUI)

Solutions were activated via ultrasonic tips (mode:E, setting:6) (DTE, Guilin Woodpecker Medical Instrument Co., Ltd., Guilin, Guangxi, China) and an ultrasonic device (DTE S6 Led, Guilin Woodpecker Medical Instrument Co., Ltd., Guilin, Guangxi, China). An ultrasonic tip one size smaller than the master apical file was positioned 2 mm short of the working length without contacting the canal walls, as described previously [10]. The canals were irrigated with 17% EDTA in three consecutive 20 s applications of 2 mL each, with fresh solution used for each period, totaling 6 mL over 1 min. Between EDTA and NaOCl irrigation, 2 mL of saline was applied for 20 s to prevent chemical interaction. The 2.5% NaOCl was then applied in three consecutive 20 s applications of 2 mL each, again using fresh solution for each period, totaling 6 mL over 1 min.

#### 2.3.5. Group 4 (PIPS)

A Fotona Er:YAG laser device (LightWalker Fotona, Ljubljana, Slovenia) was used for activation. A special conical fiber tip (PIPS 300/14, Fotona) was placed in the coronal part of the pulp chamber, and the irrigant solutions in the canal were activated in SSP mode (50 μs, 0.3 W, 15 Hz and 20 mJ) with the air and water settings turned off, as described previously [28]. The canals were irrigated with 17% EDTA in three consecutive 20 s applications of 2 mL each, with fresh solution used for each period, totaling 6 mL over 1 min. Between EDTA and NaOCl irrigation, 2 mL of saline was applied for 20 s to prevent chemical interaction. 2.5% NaOCl was then applied in three consecutive 20 s applications of 2 mL each, again using fresh solution for each period, totaling 6 mL over 1 min.

#### 2.3.6. Group 5 (SWEEPS)

A Fotona Er:YAG laser device (LightWalker Fotona, Ljubljana, Slovenia) with an 8.5 mm long and 600 µm diameter tapered fiber tip (SWEEPS 600, Fotona) was used for activation. The device was set to SWEEPS mode with two ultrashort micropulses (25 μs) continuously changing at 0.3 W, 20 mJ, and 15 Hz. The tip was placed in the pulp chamber, and the solution was activated with the air and water settings turned off, as described previously [10,28]. The canals were irrigated with 17% EDTA in three consecutive 20 s applications of 2 mL each, with fresh solution used for each period, totaling 6 mL over 1 min. Between EDTA and NaOCl irrigation, 2 mL of saline was applied for 20 s to prevent chemical interaction. 2.5% NaOCl was then applied in three consecutive 20 s applications of 2 mL each, again using fresh solution for each period, totaling 6 mL over 1 min.

After the final irrigation, the canals were dried with paper point and obturated via the cold lateral compaction method via an ADSeal (Meta Biomed, Cheongju, Republic of Korea) sealer and gutta-percha. The gutta-percha was cut 1 mm below the cemento-enamel junction, and coronal restoration was performed with composite resin (Llis, FGM, Joinville, Brazil). All procedures were performed in a single session by a single operator (M.Ç).

### 2.4. Healing Evaluation

#### 2.4.1. PAI Score and Lesion Diameter

At the 12-month follow-up, OPGs were taken using the same settings as those used for preoperative OPGs. PAI scores of the treated teeth were recorded, and patients were classified as “healed” (PAI < 3) or “unhealed” (PAI ≥ 3) [20]. PAI scoring was performed by 2 endodontists, and in cases of disagreement, a consensus was reached by discussion. Additionally, the widest diameter of the lesion at the follow-up session was measured by an endodontist (M.Ç) The same method was used for the preoperative measurements. The researchers who conducted the PAI scores and lesion size evaluations were blinded to the irrigation method and preoperative measurements.

#### 2.4.2. Fractal Analysis

**Figure 1:**
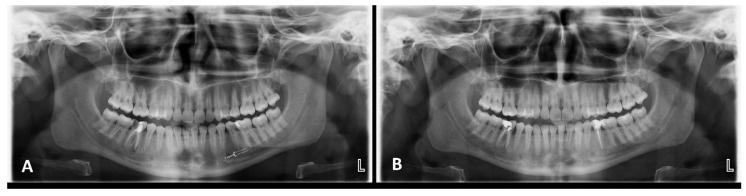
A representative example of the preoperative and 1-year follow-up radiographs for PIPS group. (**A**) Demonstration of the preoperative panoramic image of tooth #35 in the PIPS group, along with identification of the ROI used for fractal dimension analysis at the level of the root apex. (**B**) The 1-year postoperative follow-up of the periapical lesion associated with tooth #35 in the PIPS group, illustrating the healing of the periapical lesion.

Fractal dimension (FD) analysis is a quantitative method used to assess structural complexity using the box-counting algorithm [34]. FA was performed by an experienced oral and maxillofacial radiologist (D.N.G) who was blinded to the activation method and used the fractal box counting method on OPGs with ImageJ. To standardize the size and location of the region of interest (ROI), a parallel line forming a right angle to the apical and long axes of the tooth was placed 1 mm apical to the root apex (Figure 1A).

**Figure 2:**
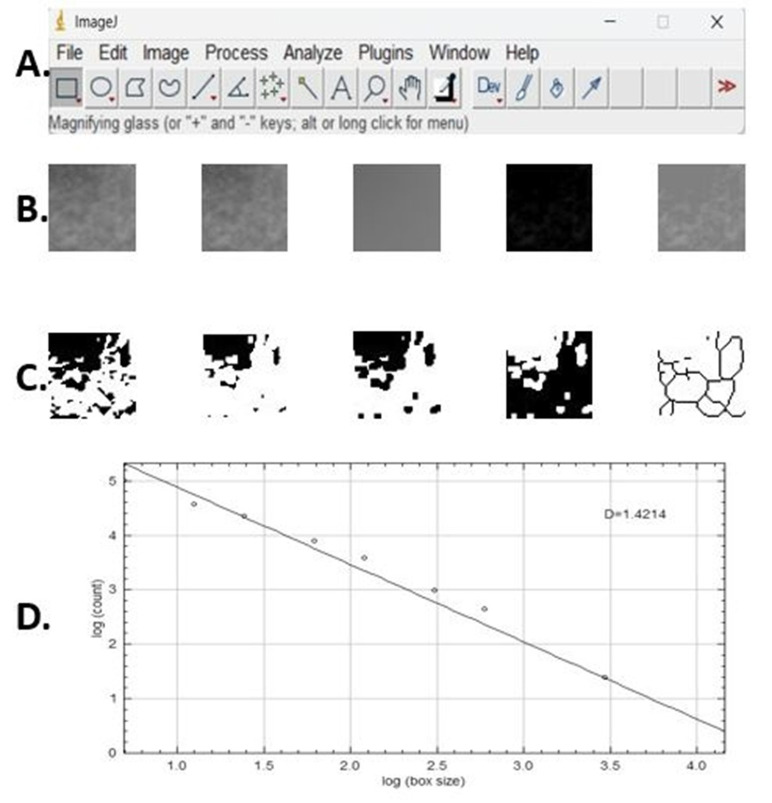
FD analysis steps: (**A**) ImageJ v. 1.52 software domain program. (**B**) From left to right: Cropped image, duplicated cropped image, blurred Gaussian image, subtraction image, and 128 added images. (**C**) From left to right: Binary image, eroded image, dilated image, inverted image, and skeletonized image. (**D**) Fractal box-counting procedure result, where the dots represent the log-transformed box counts at different box sizes and the line indicates the linear regression used to calculate the FD.

ROI selection was restricted to the periapical trabecular bone area, while the root structure, periodontal ligament space, lamina dura, cortical borders, mandibular canal/mental foramen (when visible), and superimposed anatomical structures or radiographic artifacts were deliberately excluded. The sequence of steps followed when FD analysis was performed was as follows (Figure 2): All digital images were opened in ImageJ v1.52 software, and 30 × 30 pixel ROIs were selected from predetermined areas and saved in TIFF format because of its high resolution and lossless image quality.

After the area of interest to be analyzed was cropped, it was saved in 8-bit format and copied. A Gaussian filter (sigma = 35 pixels) was applied to the duplicated image. The blurred image was subtracted from the original image via subtraction. A value of 128 was added to each pixel location, and 128 was set as the threshold value regardless of the initial brightness of the image. The 128 brightness threshold image was converted to binary format. An erosion and dilatation process was applied. The inverted image was skeletonized, and FD analysis was applied to the skeletonized image via the ‘box-counting’ function.

To assess intraobserver reliability, the lesion diameter and fractal measurements were conducted twice with 2-week intervals by the same researchers in 20% of the teeth involved in the study.

### 2.5. Statistical Analysis

The Jamovi 2.3.28 (Sydney, Australia) statistical program was used for statistical analysis. Normal distribution in the comparisons was evaluated by the Shapiro–Wilk test, Kolmogorov–Smirnov test, Anderson-Darling test and Q-Q graph. Homogeneity between variances was analyzed by Levene’s test. Since sex data were categorical, the differences between the groups were analyzed via the chi-square test. Since the distribution of age between groups was found to be normal, it was analyzed by Welch’s one-way ANOVA test.

Although a normal distribution was obtained in FA, Welch’s one-way ANOVA test was preferred for the comparison of irrigation groups because the variances were not homogeneously distributed, and pairwise comparisons were made with the Games-Howell test. Before and after comparisons within each group, paired sample *t* tests were used.

Since a normal distribution could not be obtained, the Kruskal–Wallis test was preferred for the comparison of irrigation groups for lesion size, and pairwise comparisons were made with the Dwass-Steel-Critchlow-Fligner test. Before and after comparisons within each group, the Wilcoxon rank test was used. The significance level for the statistical analysis was set to *p* < 0.05.

Additionally, intraobserver reliability was evaluated for fractal and lesion size measurements via the concordance correlation coefficient (CCC).

## 3. Results

**Figure 3:**
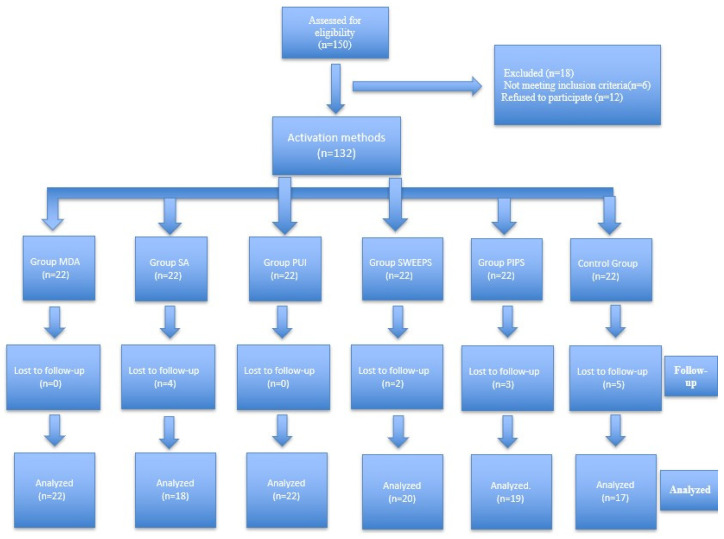
Study flow diagram.

132 patients were included in the study. However, during the follow-up, 14 patients were lost to follow-up for various reasons (including personal or logistical issues, changes in health status, or unwillingness to complete study procedures) as shown in the study flow diagram (Figure 3). During the 1-year follow-up period, a total of 118 patients were analyzed, including 22 (10 RCT, 12 RT) in the MDA group, 18 (10 RCT, 8 RT) in the SA group, 22 (11 RCT, 11 RT) in the PUI group, 20 (9 RCT, 11 RT) in the SWEEPS group, 19 (11 RCT, 8 RT) in the PIPS group, and 17 (9 RCT, 8 RT) in the control group. Among these, 55 were females (46.6%) and 63 were males (53.4%). The baseline demographic and clinical characteristics for each group are shown in Table 1.

### 3.1. Radiographic Assessment of Lesion Diameter

The lesion diameter significantly decreased with each activation method at the 1-year follow-up (*p* < 0.001); however, no significant decrease was observed in the control group (*p* > 0.05) (Table 2). No significant difference was observed in preoperative lesion diameter between the groups (*p* > 0.05). At the 1-year follow-up, the lesion diameter was significantly smaller in the PIPS and SWEEPS groups than in the control group (*p* = 0.002). No significant difference was detected in the other group comparisons (*p* > 0.05) (Table 2).

### 3.2. Assessment of PAI-Based Healing

Prior to RCT, no statistically significant differences were observed in PAI scores among the irrigation groups (*p* > 0.05). However, following treatment, a significant reduction in PAI scores was detected for all groups each irrigation device compared with the corresponding preoperative values (*p* < 0.001). The postoperative decrease in PAI scores was significantly greater in the PIPS and SWEEPS groups than in the control group (*p* < 0.05) (Table 3).

Moreover, when cases were categorized according to PAI evaluation as healed (PAI < 3) or not healed (PAI ≥ 3) (12), the lowest healing rate was observed in the non-activated control group (41.1%), whereas the PIPS (100%) and SWEEPS (95%) groups exhibited the highest healing outcomes at the 1-year follow-up (Table 4). Although the control group showed the lowest percentage of healing, this finding does not necessarily indicate a lack of lesion healing. For example, a PAI score of 5 before treatment may have decreased to 3 after treatment; however, since classification was based on categorical cutoffs, such cases were still considered “not healed.” Nevertheless, it should be noted that postoperative PAI scores decreased in all groups.

### 3.3. Fractal Dimension (FD) Analysis

In all groups, the FD was significantly greater at the 1-year follow-up than at baseline (*p* < 0.001). Both the preoperative and 1-year follow-up FD values in the SA group were significantly greater than those in the SWEEPS group (*p* < 0.05), whereas there was no significant difference between the other groups (*p* > 0.05). Additionally, no significant difference was found between the groups in terms of the degree of increase in FD (*p* > 0.05) (Table 5).

As a result of the intraobserver reliability analysis of the fractal and lesion size measurements, the CCC values were calculated as 0.836 (95% CI: 0.672–0.922) and 0.810 (95% CI: 0.645–0.902), respectively, and the measurements were found to be highly reproducible and reliable.

No adverse events were observed in any of the study groups.

## 4. Discussion

Although many studies in the literature have compared many characteristics of activation methods, such as debris removal, antimicrobial efficacy and effective activation capacity, few studies have evaluated the effects of these methods on the healing of apical lesions [25,26,27,28,29,30,35]. This study evaluated the effects of different irrigation methods on lesion healing in teeth with CAP. Although no superiority was found over CSI in terms of trabecular healing pattern, irrigation activation, particularly PIPS and SWEEPS, was found to be significantly effective in reducing the diameter of periapical lesions at 1-year follow-up after endodontic treatment.

Systemic diseases may have a negative effect on the success of endodontic treatment [36]. In studies comparing individuals with systemic diseases that negatively affect bone formation and destruction with healthy individuals, significant differences were detected in FD values [37,38,39]. Therefore, patients without systemic diseases were included in this study.

The PAI was originally described for use with periapical radiographs (PR) [40]. However, in some studies, it has also been applied using OPGs [41,42] or a combination of OPGs and PRs for the detection of AP [43]. In a systematic review published in 2024 [44] evaluating the diagnostic accuracy of PR and OPG in the detection of AP, it was recommended that PR be used for the analysis of periapical lesions located in the maxillary and mandibular incisor regions due to differences in accuracy across specific anatomical areas. In contrast, mandibular premolar and molar regions were reported to be assessed with comparable accuracy using either PR or OPG. Moreover, when focusing on the diagnostic accuracy of OPG, higher accuracy was observed in the mandibular canine and premolar regions compared with maxillary regions. PR and OPG showed similar diagnostic accuracy for both endodontically treated and untreated teeth; however, OPG yielded slightly better results [44]. This finding suggests that previous endodontic treatment may influence the visualization and detection of AP lesions. It was emphasized that mandibular premolar and molar regions can be evaluated interchangeably using PR or OPG, and that two-dimensional imaging should be considered a first-line examination method for the identification of periapical lesions and treatment planning in all cases [44]. Based on the findings of these studies, mandibular premolar teeth were selected in the present study, and PAI scores were assessed using OPGs.

In the literature, many studies have reported that the preoperative size of periapical lesions can affect the outcome of periapical healing [20,45,46,47]. Lesions smaller than 5 mm have been associated with significantly better healing [45], whereas increased lesion severity may compromise treatment success [46]. In addition, studies have shown that teeth with higher baseline PAI scores tend to have lower healing rates [20,47]. Therefore, in our study, the similarity of baseline PAI scores and lesion sizes between groups strengthens the interpretation that the observed differences in healing are attributable to the treatment itself.

In this study, laser groups showed significantly higher healing compared to the CSI group, in terms of PAI scores and lesion diameter. A representative example of the pretreatment and 1-year follow-up radiographs for PIPS group is shown in Figure 1B. On the basis of these results, the null hypothesis of this study was rejected. In a systematic review published in 2024 evaluating the effects of lasers on the healing of periapical lesions, it was concluded that laser systems accelerate and enhance bone healing in the periapical region [48]. Achieving complete chemical debridement of the root canal is highly important in the healing process after endodontic treatment. Laser activation techniques have been shown to enhance the removal of pulpal tissue residues, smears and debris by delivering the solution to hard-to-reach areas [26,27]. Additionally, it has been reported to be highly effective in eliminating bacterial biofilms from infected root canals [13]. This high cleaning efficiency may explain the significant lesion reduction in the laser groups compared with that in the CSI group in the present study. Similarly to the present study, Uslu et al. reported that neither PIPS nor SWEEPS was superior to the other in terms of smear layer removal [30]. In addition, another study revealed that both laser applications showed similar efficacy in eliminating E. faecalis, the most frequently isolated bacterium in endodontic treatment failures [29]. However, contrary to these findings, some in vitro studies have demonstrated that SWEEPS is more effective than PIPS in debris removal [28,49]. In one of these in vitro studies, the amount of residual debris was evaluated in different regions of the root canals of mandibular molar teeth [49], whereas in the other study, the remaining debris was assessed in straight single-rooted mandibular premolar teeth [28]. The differing results between studies may be attributed to several factors, including differences in sample size, study design, tooth type and the anatomy of the evaluated region (apical, coronal, or middle third), as well as variations in the IATs used.

In the present study, activation groups other than the laser group were not superior to the CSI group. Similarly, in a randomized controlled clinical trial conducted in single-rooted teeth with AP, periapical healing was reported to be 95.1% in the ultrasonically activated group and 88.4% in the CSI group, with no significant difference [50].

In another study evaluating lesion healing of mandibular single-rooted teeth volumetrically with Cone Beam Computed Tomography (CBCT), no significant difference was detected between the SWEEPS, PUI, MDA and CSI groups at the 1-year follow-up [10]. In an in vitro study comparing the effectiveness of SA and CSI in removing the smear layer and debris in curved canals of permanent molars, no statistically significant difference was found [51].

Detecting new bone formation in the periapical region of teeth with AP after endodontic treatment is very important for evaluating treatment success [24]. FA is increasingly used to assess bone healing and structural changes in clinical conditions, such as AP and endodontic treatments. It provides quantitative, reliable, and objective information on trabecular bone patterns and is not influenced by projection geometry or radiodensity [52,53]. In this study, healing was also evaluated with FA via the box-counting method. A high FD value indicates a more complex structure with fewer intertrabecular spaces, whereas a low FD value indicates a simple structure with more intertrabecular spaces [54]. Previous studies have reported significant increases in FD values following successful RCT or RT procedures [23,55]. In a study examining the effects of regenerative endodontic treatment on bone structure using OPG and FA, both a decrease in PAI scores and an increase in FD values were observed [22]. In line with these studies, in this study the 1-year follow-up FD values were significantly greater in all groups than in the baseline data for each activation method and the CSI group. Also, both preoperative and follow-up FD values in the SA group were found to be significantly higher than those in the SWEEPS group. However, since the preoperative FD values in the SA group were already higher than those of SWEEPS, the follow-up difference alone is not sufficient to demonstrate the superiority of SA over SWEEPS.

In this study, although laser activation significantly improved lesion shrinkage and PAI outcomes compared with the other irrigation protocols, no significant difference was observed among the groups in terms of FD increase. In other words, a central increase in trabeculation occurred due to an increase in bone density in all irrigation groups, with no superiority to each other. This indicates increased complexity in bone structure, suggesting healing or ongoing recovery [24]. Similarly to the current study, Tosun et al. evaluated the trabecular pattern of teeth with AP 1 year after RT via FA and PAI. The authors reported a success rate of 70% according to the PAI and noted that FD significantly increased in healed patients (PAI < 3) and decreased in unhealed patients (PAI ≥ 3). They also reported no significant correlation between the PAI and changes in FD [56].

Radiographic lesion healing and changes in trabecular bone structure (FD change) may reflect different aspects of the same biological healing process [21,24,56,57,58]. This is because fractal analysis is sensitive to biological variability. In other words, trabecular bone structure is influenced not only by bone density but also by processes such as demineralization, new bone formation, and trabecular reshaping [57]. For example, in chronic periapical periodontitis and cysts, during the increased demineralization process, trabecular patterns may become more visible, leading to higher FD values despite a decrease in bone density [21]. Conversely, the connectivity of large trabecular supports may reduce trabecular density, which can result in a decrease in FD [57,59]. In the study by Yu et al., unlike others, FA was applied to the reactive bone area instead of the radiolucent area and a significant decrease in FD values was reported at the 6-month follow-up after clinically successful endodontic treatment, supporting the change in dense reactive bone toward normal density [58]. This variability limits the use of FA as an independent diagnostic tool and emphasizes the need for complementary radiographic and clinical assessments for accurate interpretation [57,60]. Significant increases in FD values have been associated with enhanced trabecular formation during the healing process. These findings are further supported by the marked reduction in lesion size and corresponding PAI scores over time, consistent with other studies in the literature [22,56,57]. In light of these findings, it may be suggested that trabecular remodeling and lesion resolution represent distinct biological processes and that laser activation can accelerate lesion resolution without altering trabecular complexity.

The strengths of this study include its prospective, follow-up clinical design, the use of advanced laser activation techniques and its relatively large sample size. However, there are some limitations of this study.

Both primary RCT and RT cases were included in the study. This represents a potential confounder. Although their distribution across the groups was relatively balanced, the inherent clinical differences between these treatment modalities, such as prior treatment history, anatomical complexity, and potential tissue response, may have contributed to some degree of heterogeneity [61]. Nevertheless, a retrospective study comparing periapical lesion healing in teeth with CAP treated with primary RCT and RT using FA reported no significant difference in healing rates between the two treatment types [23], suggesting that the inclusion of both modalities in our study may have had a limited impact. However, future studies evaluating RCT and RT in separate groups would help better control for potential confounding effects.

Quasi-randomization was used to achieve a balanced distribution of RCT and RT cases across the study groups while maintaining a practical allocation process during patient recruitment. However, because the allocation sequence may be predictable, this method may increase the risk of selection bias compared with true randomization. Nevertheless, some evidence suggests that quasi-randomized trials do not necessarily show greater baseline imbalance than fully randomized trials [62]. This method has also been used in endodontic clinical research evaluating sodium hypochlorite irrigation protocols [63,64]. Therefore, although the absence of true randomization may represent a methodological limitation, quasi-randomization can still provide balanced group allocation and practical feasibility in clinical settings.

It has been demonstrated that CBCT shows higher diagnostic accuracy in the detection of periapical lesions compared with PR and OPG [65]. Considering the patient profile in the present study, two-dimensional radiographic evaluation was preferred over routine CBCT imaging due to dosimetric considerations [44]. In the present study, FA was performed on OPGs because of its greater convenience, easier application method and lower radiation dose. However, OPGs are two-dimensional and do not provide the opportunity to evaluate the effect of the lesion on the cortical bone in the buccolingual region.

Future studies could incorporate CBCT-based volumetric assessments to provide a more precise evaluation of periapical lesion healing, particularly in the buccolingual dimension, which cannot be fully assessed with two-dimensional OPG or PRs. Moreover, longer follow-up periods (>12 months, e.g., 24 months) are recommended to confirm long-term healing stability and detect delayed complications. Such approaches would complement the current findings and strengthen the clinical relevance of future research.

## 5. Conclusions

Within the limitations of this study, lesion diameters decreased significantly at the 1-year follow-up in all the activation groups. Laser activation (PIPS and SWEEPS) improved lesion reduction and PAI outcomes compared with CSI. Also, FD increased in all groups without significant intergroup differences. These findings suggest that laser activation may enhance lesion healing; however, further randomized clinical studies with longer follow-up periods and volumetric imaging are needed to confirm these results.

## Figures and Tables

**Table 1:** Demographic and clinical characteristics for each group.

Group	n	Mean Age (Range)	Female/Male (n)	RCT/RT (n)
MDA	22	44.0 (18–73)	7/15	10/12
PUI	22	44.7 (21–65)	11/11	11/11
SA	18	48.8 (20–70)	4/14	10/8
PIPS	19	43.2 (19–63)	10/9	11/8
SWEEPS	20	44.8 (26–74)	12/8	9/11
CONTROL	17	53.2 (25–71)	11/6	9/8
Total	118	46.2 (18–74)	55/63	60/58

RCT: Root canal treatment/RT: Retreatment.

**Table 2:** The effect of root canal treatment and irrigation activation techniques on lesion diameter.

	Preoperative		1-Year Follow-Up		
	Mean ± SD	Median (Min–Max)	Mean ± SD	Median (Min–Max)	*p*-Value
Control	3.01 ± 1.21 ^a^	2.6 (1.3–5.3) ^a^	2.07 ± 2.28 ^a^	1.2 (0.2–8) ^a^	0.052 ^1^
SA	3.11 ± 1.42 ^a^	2.8 (1.6–6.1) ^a^	1.12 ± 1.84 ^ab^	0.3 (0.1–6.8) ^ab^	0.001 ^1^
MDA	2.79 ± 1.67 ^a^	2.3 (1–7.5) ^a^	0.98 ± 1.65 ^ab^	0.5 (0.1–7.8) ^ab^	<0.001 ^1^
PIPS	2.96 ± 1.28 ^a^	2.5 (1.1–6.4) ^a^	0.33 ± 0.24 ^b^	0.3 (0.1–0.9) ^b^	<0.001 ^1^
PUI	3.17 ± 2.04 ^a^	2.45 (1.2–8.5) ^a^	0.78 ± 1.58 ^ab^	0.4 (0.1–7.7) ^ab^	<0.001 ^1^
SWEEPS	2.43 ± 0.94 ^a^	2.05 (1.2–4.8) ^a^	0.43 ± 0.64 ^b^	0.2 (0.1–3) ^b^	<0.001 ^1^
*p*-value	0.569 ^2^		0.002 ^2^		

^1^ Wilcoxon rank test, ^2^ Kruskal–Wallis test, different letters in the same column indicate significant difference according to Dwass-Steel-Critchlow-Fligner pairwise comparison test (*p* < 0.05).

**Table 3:** The effect of root canal treatment and irrigation techniques on PAI scores.

	Preoperative		1-Year Follow-Up		
	Mean ± SD	Median (Min–Max)	Mean ± SD	Median (Min–Max)	*p*-Value
CONTROL	4.412 ± 0.507 ^a^	4 (4–5) ^a^	2.647 ± 1.272 ^a^	3 (1–5) ^a^	<0.001 ^1^
SA	4.056 ± 0.802 ^a^	4 (3–5) ^a^	1.778 ± 1.215 ^ab^	1 (1–5) ^ab^	<0.001 ^1^
MDA	4.136 ± 0.834 ^a^	4 (3–5) ^a^	2.000 ± 0.976 ^ab^	2 (1–5) ^ab^	<0.001 ^1^
PIPS	4.211 ± 0.787 ^a^	4 (3–5) ^a^	1.421 ± 0.507 ^b^	1 (1–2) ^b^	<0.001 ^1^
PUI	4.318 ± 0.568 ^a^	4 (3–5) ^a^	1.864 ± 0.941 ^ab^	2 (1–5) ^ab^	<0.001 ^1^
SWEEPS	4.100 ± 0.641 ^a^	4 (3–5) ^a^	1.400 ± 0.754 ^b^	1 (1–4) ^b^	<0.001 ^1^
*p*-value	0.710 ^2^		0.003 ^2^		

^1^ Wilcoxon rank test, ^2^ Kruskal-Wallis test, different letters in the same column indicate significant difference according to Dwass-Steel-Critchlow-Fligner pairwise comparison test (*p* < 0.05).

**Table 4:** Distribution of ‘healed’ and ‘unhealed’ lesions at 1-year-follow-up according to PAI score.

Irrigation Methods	Healed (PAI < 3)	Unhealed (PAI ≥ 3)	Healing Percentage
MDA	17	5	77.2%
SA	14	4	77.7%
PUI	19	3	86.3%
PIPS	19	0	100%
SWEEPS	19	1	95%
CONTROL	7	10	41.1%

**Table 5:** The effect of root canal treatment and irrigation techniques on fractal dimension.

	Preoperative		1-Year Follow-Up			Increase in Fractal Dimension
	Mean ± SD	Median (Min–Max)	Mean ± SD	Median (Min–Max)	*p*-Value	Mean ± SD
Control	1.216 ± 0.044 ^ab^	1.212 (1.127–1.287) ^ab^	1.326 ± 0.046 ^ab^	1.325 (1.21–1.386) ^ab^	<0.001 ^1^	0.110 ± 0.066 ^a^
SA	1.25 ± 0.034 ^a^	1.253 (1.181–1.306) ^a^	1.345 ± 0.025 ^a^	1.341 (1.311–1.39) ^a^	<0.001 ^1^	0.095 ± 0.038 ^a^
MDA	1.23 ± 0.04 ^ab^	1.222 (1.172−1.319) ^ab^	1.338 ± 0.035 ^ab^	1.327 (1.285−1.395) ^ab^	<0.001 ^1^	0.108 ± 0.044 ^a^
PIPS	1.249 ± 0.042 ^ab^	1.251 (1.162−1.311) ^ab^	1.322 ± 0.038 ^ab^	1.319 (1.236–1.391) ^ab^	<0.001 ^1^	0.073 ± 0.045 ^a^
PUI	1.235 ± 0.049 ^ab^	1.23 (1.123–1.302) ^ab^	1.311 ± 0.055 ^ab^	1.314 (1.213–1.391) ^ab^	<0.001 ^1^	0.076 ± 0.048 ^a^
SWEEPS	1.216 ± 0.025 ^b^	1.217 (1.16–1.253) ^b^	1.3 ± 0.057 ^b^	1.315 (1.19–1.394) ^b^	<0.001 ^1^	0.084 ± 0.051 ^a^
*p*-value	0.009 ^2^		0.016 ^2^			0.104 ^2^

^1^ Paired-Simple *t*-test, ^2^ Welch’s One-way ANOVA test, different letters in the same column indicate significant difference according to Games-Howell pairwise comparison test (*p* < 0.05).

## Data Availability

The data that support the findings of this study are available from the corresponding author upon reasonable request.

## Supplementary Materials

The following supporting information can be downloaded at: https://www.mdpi.com/article/10.3390/diagnostics16071003/s1, Supplementary Table S1: TREND Statement Checklist [66].

## Abbreviations

IAT	Irrigation Activation Technique
AP	Apical Periodontitis
CAP	Chronic Apical Periodontitis
PAI	Periapical Index
CSI	Conventional Syringe Irrigation
SA	Sonic Activation
PUI	Passive Ultrasonic Irrigation
PIPS	Photon-Induced Photoacoustic Streaming
SWEEPS	Shock Wave Enhanced Emission Photoacoustic Streaming
MDA	Manual Dynamic Activation
FD	Fractal Dimension
FA	Fractal Analysis
OPG	Panoramic Radiography
PR	Periapical Radiography
RCT	Root Canal Treatment
RT	Retreatment
CCC	Concordance Correlation Coefficient
CBCT	Cone Beam Computed Tomography
